# Quality of life in Slovenian patients with skull base tumours: cross-cultural adaptation and validation of a Slovenian skull base inventory

**DOI:** 10.2478/raon-2026-0007

**Published:** 2026-01-21

**Authors:** Domen Vozel, Jure Urbancic, Saba Battelino, Nina Bozanic Urbancic, Nejc Steiner, Tomislav Felbabic, Roman Bosnjak

**Affiliations:** Department of Otorhinolaryngology and Cervicofacial Surgery, University Medical Centre Ljubljana, Ljubljana, Slovenia; Faculty of Medicine, University of Ljubljana, Ljubljana, Slovenia; Manchester University NHS Foundation Trust, Wythenshawe Hospital, Southmoor Rd, Wythenshawe, Manchester, Great Britain; Department of Neurosurgery, University Medical Centre Ljubljana, Ljubljana, Slovenia

**Keywords:** quality of Life, skull base, neoplasms, meningioma, carcinoma

## Abstract

**Background:**

Skull base tumours frequently manifest as severe physical morbidity and quality of life (QoL) impairment. The disease-related QoL measurement can be performed with disease specific questionnaires, e.g. Skull base inventory (SBI).

**Patients and methods:**

The study consisted of two parts: (1) cross-cultural adaptation and psychometric testing of the Slovenian SBI (SBI-SLO) and (2) QoL assessment in skull base tumours. Two groups completed the SBI-SLO: 1.) adult patients without prior treatment of anterior, anterolateral and/or central skull base and 2.) healthy controls. Patients with skull-base tumours were further analysed for difference in SBI-SLO total score and domain scores between 1.) benign and malignant tumours and 2.) pituitary macroadenomas and other benign tumours.

**Results:**

59 patients (46% male, 54% female, median age 57.7 years) and 47 subjects from control group (49% male, 51% female, median age 42,2 years) completed SBI-SLO, which demonstrated an excellent level of internal consistency (Cronbach’s alpha = 0.924) and excellent test-retest reliability (intraclass correlation coefficient [ICCA] = 0.952). The discriminant validity was confirmed (*p* = 0.000). SBI-SLO total score, emotional, other and family domain scores were lower in malignant than in benign tumours (*p* = 0.031, *p* = 0.038, *p* = 0.008, and *p* = 0.046, respectively). Macroadenoma and other benign tumours differed only in neurological domain score (p < 0.05).

**Conclusions:**

A skull base tumour, especially malignant, can exert a substantial detrimental effect on a patient’s quality of life. The SBI is a key tool for assessing QoL, also available in Slovenian.

## Introduction

The skull base is the anatomical area that separates the intracranial and extracranial spaces. It is notable for its inclusion of significant neurovascular structures, including all cranial nerves, the internal carotid and vertebral arteries, and the brain stem. On the extracranial surface, the skull base is bordered by the nose and paranasal sinuses, the nasopharynx, the orbit, and the middle and inner ear. On the intracranial surface, it is adjacent to the pituitary gland, cerebrum, cerebellum and brain stem.

Skull base tumours frequently manifest as severe morbidity due to damage to these structures, causing significant impairment of organ function and quality of life. Research has demonstrated that, following treatment for a skull base tumour, organ function recovers more rapidly than quality of life. Consequently, the quality of life is a pivotal metric for evaluating the efficacy of treatment modalities for skull base tumours.^1^

The measurement of health-related quality of life (HRQoL) can be facilitated through the utilisation of various patient-reported outcome measures (PROMs), most commonly in the form of questionnaires. Multiple questionnaires have been developed to measure the quality of life concerning skull base disease. These include the Skull base inventory (SBI)^2^, the Anterior skull base questionnaire (ASBQ)^3^ and the Sino-nasal outcome test for neurosurgery (SNOT-NC).^4^ The SBI encompasses both physical and non-physical domains for the assessment of quality of life, and its utilisation is appropriate for patients both before and after endoscopic and open procedures of the anterior, anterolateral or central skull-base.^2,5^

According to the WHO definition, quality of life means: “*individuals’ perception of their position in life in the context of their culture, and value systems in which they live and in relation to their goals, expectations, standards and concerns*”.^6^ This implies that each PROM in the form of a questionnaire should be adapted to the language used by the patient. To assess quality of life in patients with skull base tumours in the Slovenian population, a suitable Slovenian-language questionnaire was required.

This article therefore presents the cross-cultural adaptation and validation of the Slovenian version of the SBI (SBI-SLO), which was used to assess quality of life in patients with anterior, anterolateral and central skull base tumours managed at a tertiary referral centre. The study aimed to develop a validated and culturally adapted tool for evaluating disease- or anatomical area-specific quality of life in patients with skull base disease in Slovenia, and to identify differences in quality of life between those with benign and malignant tumours.

## Patients and methods

**FIGURE 1.**
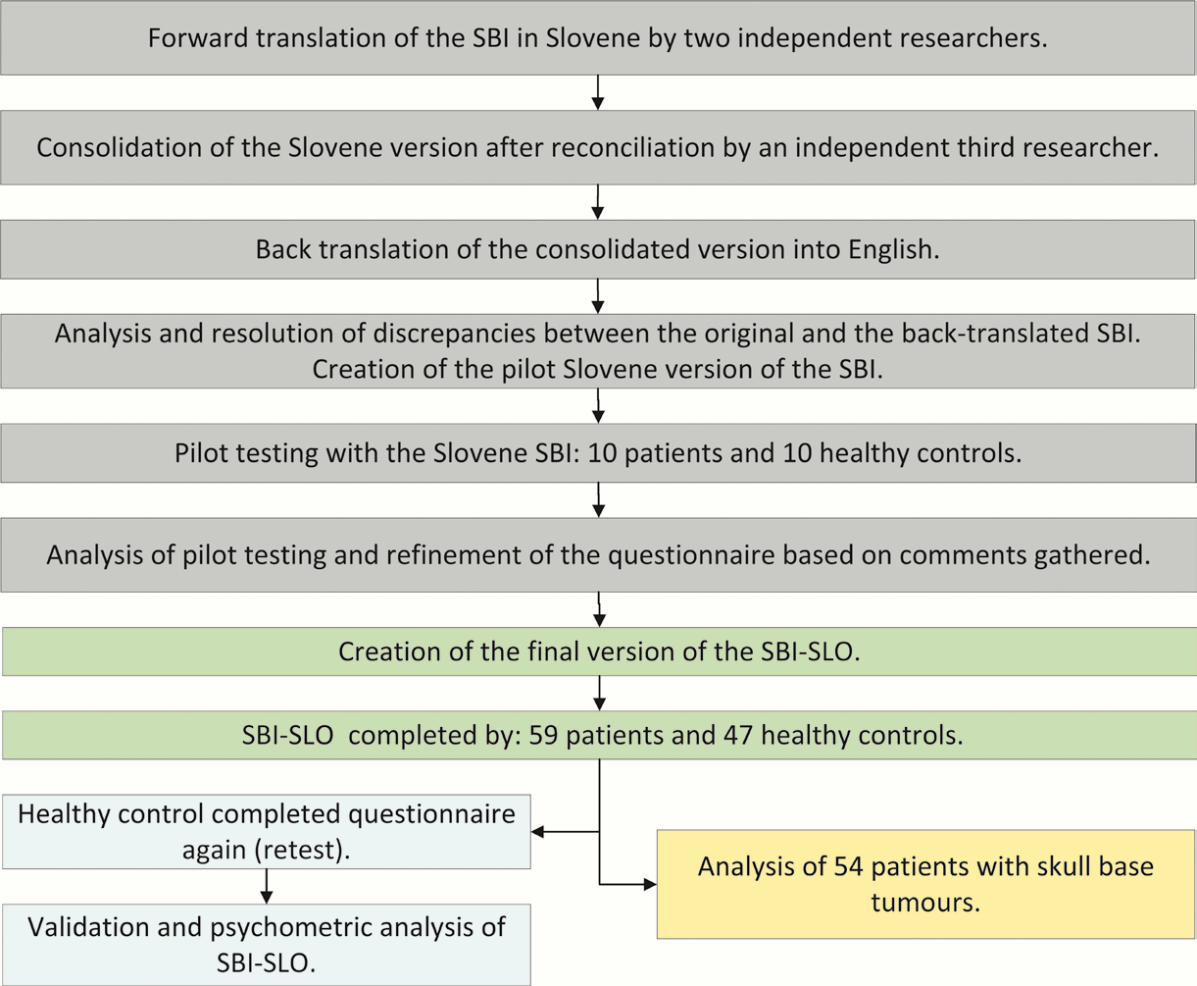
Workflow diagram of the study. SBI = skull base inventory; SBI-SLO = Slovenian version of SBI

The study consisted of two arms: 1.) cross-cultural adaptation and psychometric testing of the SBI-SLO and 2.) quality of life assessment in skull base tumours as depicted in Figure 1.

National Medical Ethics Committee approved the study (0120-425/2023/4, November 22^nd^, 2023), which was registered on www.clinicaltrials.gov (NCT05607888). All subject in this study provided an informed consent.

### Development of the Slovenian version of skull base inventory (SBI-SLO)

Development of Slovenian version of SBI (SBI-SLO) consisted of the following chronologically order of process: translation, cross-cultural adaptation and psychometric testing as described in the following manuscript.

#### Translation and cross-cultural adaptation

After conducting a literature review, we did not identify a Slovenian version of SBI. Cross-cultural adaptation and validation of the SBI-SLO was performed according to the methodology described by Hall *et al*.^7^

Permission to use the original SBI was obtained from the corresponding author of the relevant publication on May 18^th^, 2021.^2^ Relevant attributes, such as literacy, population characteristics and the requirement for administrative help, were evaluated for the target population consisting of patients with skull base disease.

First the original version^2^ was translated independently in Slovenian language by two bilingual researchers (DV and NS) (i.e., forward translation). Discrepancies were reconciled by a third independent researcher (SB), and a single consolidated version of questionnaire was created. Then, two Slovenian and English proficient researchers (NBU and JU) translated the consolidated version back into an English version (i.e., back-translation). Discrepancies between the original version and back translated version were reported and solved by a multidisciplinary team, which included Slovenian researchers speaking fluent English as a foreign language (DV, NS, SB, JU, NBU, TF, RB). A pilot version of the Slovenian SBI was then created.

A pilot testing was performed by administering the pilot version of SBI-SLO to the target audience (i.e., ten subjects from each group described below). After the pilot testing the comments were gathered. Then the final version of SBI-SLO was created.

#### Slovenian version of skull base inventory questionnaire

SBI-SLO consisted of 41 questions across eleven domains (cognitive, emotional, family, financial, social, spiritual, endocrine, nasal, neurologic, visual and other domain) (Supplementary data 1). Scores were calculated in the same way as in original questionnaire (Supplementary data 2). Each item is answered on a 7-point scale, where the answer 0 means the worst situation and answer 6 means the best situation. The domain score was calculated as ([sum of points for that domain / maximum possible points] × 100). A score of 0 means the worst possible quality of life, and 100 the best. The total score is calculated across all 11 domains, each contributing 1/11, so the overall possible range is 0–100.^2^

#### Subjects

Two independent groups with the following inclusion criteria completed the SBI-SLO:

a) Patients group:
  - age at least 18 years,
  - active knowledge of the Slovene language
  - no prior treatment of the skull-base disease,
  - disease that involves any part or layer of the anterior, anterolateral and/or central skull base,
  - diagnosis confirmed pathohistologically and/or radiologically
  - disease managed at the at University Medical Centre Ljubljana.
b) Control group:
  - age at least 18 years,
  - active knowledge of the Slovene language,
  - healthy

The control group completed the questionnaire again at least one day later (i.e., control retest group) for the purpose of test-retest reliability analysis.

The exclusion criteria were:

- did not meet inclusion criteria,
- disease centred in the lateral skull base (e.g., cholesteatoma, vestibular schwannoma, paraganglioma),
- prior treatment of skull-base disease.

#### Statistical analysis and psychometric testing

After the translation and cross-cultural adaptation, the psychometric testing was performed using various statistical methods; these are thoroughly described to enable replicability. Data were analysed using Microsoft Excel for Mac (version 16 and later) and SPSS (Statistical Package for the Social Sciences, version 23, IBM Corp., Armonk, NY, USA).

Data was reported as a mean +/- standard deviation in normally distributed data and as a median with range in non-normally distributed data. Distribution normality was determined according to the Shapiro-Wilk test if the sample size was less than 50. Significant outliers were determined by assessing boxplots. Values greater than 1.5 box-lengths from the edge of the box were considered as significant outliers. In non-normally distributed data or presence of outlier(s) non-parametric statistical analysis test was utilized. *p*-value less than 0.05 was considered as statistically significant.

Internal consistency, test-retest reliability, discriminant validity and floor and ceiling effects analyses were performed for psychometric testing of SBI-SLO.

Internal consistency was assessed by the Cronbach’s alpha analysis in the patients group. Value below 0.7 was considered as an unacceptable, 0.7-0.9 as good (8)and above 0,9 as excellent internal consistency.

Test-retest reliability was assessed by type A intraclass correlation coefficient (ICC_A_) estimates and their 95% confident intervals using an absolute agreement definition on control group and control retest group. Value above 0.7 was considered as an acceptable.^8^

Discriminant validity was assessed by analysis of difference in total questionnaire scores between patient and control group.

Floor and ceiling effects were analysed for domain scores and total SBI-SLO score for the patients group as defined by a 15% threshold. If more than 15% of respondents achieved the lowest or highest possible score, they reached the floor or ceiling, respectively.^9^

### Quality of life assessment in skull base tumours

Quality of life in skull base tumours was analysed in the subgroup of patients with skull base tumours included for cross-cultural adaptation and psychometric testing of SBI-SLO. Comparisons of SBI-SLO total score and domain scores were performed for:

a) benign *vs* malignant tumours,
b) pituitary macroadenomas *vs* other benign skull-base tumours (non-macroadenomas).

## Results

### Slovenian version of Skull base inventory (SBI-SLO)

SBI-SLO was completed by 59 patients (46% male, 54% female, median age 57.7 years) and 47 subjects from control group (49% male, 51% female, median age 42.2 years).

All subjects (100%) from control group (i.e., control retest group) completed the questionnaire again after a median interval of 4 days. The SBI-SLO demonstrates an excellent level of internal consistency (Cronbach’s alpha = 0.924) and an excellent test-retest reliability (ICC_A_ = 0.952; 95% CI = 0.915-0.973). According to the Mann-Whitney U test, the discriminant validity for SBI-SLO is confirmed (*U* = 555.000, *z* = -5.288, *p* = 0.000). Inter item and item total correlations are available in Supplementary data 3.

There was a ceiling effect detected in six (54.5%) of the domains, including cognitive (18.6% of participants endorsing maximum score), family (27.1%), financial (23.7%), spiritual (25.4%), endocrine (20.3%) and visual (16.9%). No floor effects were detected.

### Skull base tumour-related quality of life

Within the patients group, 54 patients (91.5%) suffered from tumour (Table 1): 35 patients (64.8%) with a benign (57.1% females, 42.9% males) and 19 (35.2%) with a malignant tumour (52.5% females; 47.4% males). Other 5 patients (8.5%) suffered from infection (4) or inflammation (1). There were no statistically significant differences in gender distribution (*p* > 0.05, test of two proportions) and median age (*p* > 0.05, Mann-Whitney U test) between benign (54.9 years) and malignant tumours (67.4 years).

**Table 1.** Tumour types in the studied group

Tumour type	n = 54	%
Pituitary macroadenoma^b^	17	31.5
Sinonasal cancer^m^	12	22.2
Nasopharyngeal cancer^m^	4	7.4
Olfactory meningioma^b^	3	5.6
Tuberculum sellae meningioma^b^	3	5.6
Metastasis^m^	2	3.7
Petroclival meningioma^b^	2	3.7
Sellar meningioma^b^	2	3.7
Sphenoid sinus meningoencephalocele^b^	2	3.7
Clival chordoma^m^	1	1.9
Craniopharyngioma^b^	1	1.9
Infratemporal fossa schwannoma^b^	1	1.9
Optic nerve germinoma^m^	1	1.9
Rathke cleft cyst^b^	1	1.9
Sellar teratoma^b^	1	1.9
Skull base hemangioma^b^	1	1.9

b = benign tumour;

m = malignant tumour

**FIGURE 2.**
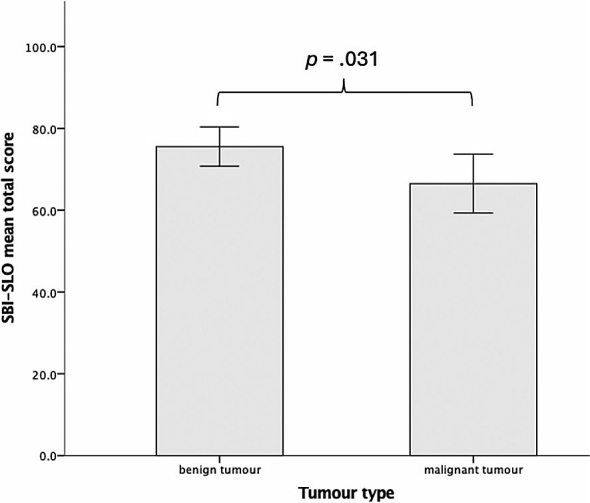
Simple bar chart depicting the differences in Slovenian version of skull base inventory (SBI-SLO) mean total score between benign and malignant tumour group.

An independent-samples t-test was run to determine if there were differences in SBI-SLO total score between patients with benign and malignant tumours. The SBI-SLO total score was lower in group of malignant (66.5 ± 15.0) than in benign tumours (75.6 ± 14.0), a statistically significant difference of 9.1 (95% CI, 0.9 to 17.3), *t*(52) = 2.220, *p* = 0.031, *d* = 0.91 (Figure 2).

**FIGURE 3.**
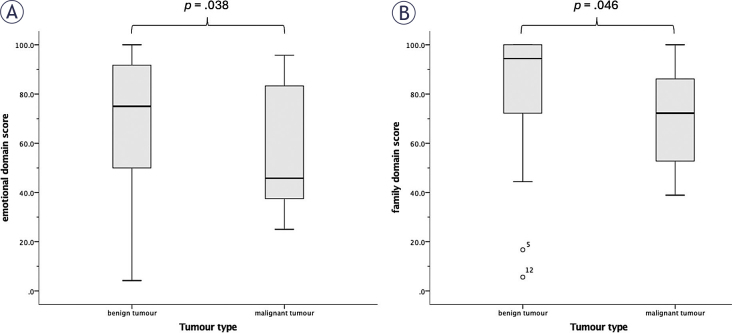
Boxplots of comparisons between patients with benign and malignant skull base tumours in emotional **(A)** and family domain score **(B)** of Slovenian version of Skull base inventory.

Emotional, family and other domain scores of SBI-SLO were lower in malignant than in benign tumours group (Figure 3), a statistically significant difference assessed by Mann-Whitney U test (p = 0.038, *p* = 0.046 and *p* = 0.008, respectively). Cognitive, financial, social, spiritual, endocrine, nasal, neurologic and visual domain scores were not statistically significantly different between benign and malignant tumour group (*p* > 0.05) (Table 2).

**TABLE 2.** Differences in scores of Slovenian version of Skull base inventory between patients with benign and malignant skull base tumours

	Benign tumour group (*n* = 35)	Malignant tumour group (*n* = 19)	*p*-value
**Total score**	75.6 ± 14.0	66.5 ± 15.0	*p* = .031*
cognitive	83.3	75.0	*p* = .102**
emotional	75.0	45.8	*p* = .038**
family	94.4	72.2	*p* = .046**
financial	83.3	66.7	*p* = .058**
social	65.4 ± 23.4	61.8 ± 21.7	*p* = .591*
spiritual	91.7	83.3	*p* = .329**
endocrine	87.5	87.5	*p* = .862**
nasal	67.3 ± 20.5	55.0 ± 24.5	*p* = .056*
neurologic	75.0	62.5	*p* = .125**
visual	83.3	70.8	*p* = .075**
other	75.0	58.3	*p* = .008**

* = independent samples t-test;

** = Mann-Whitney U test; p = p-value

1 Statistically significant differences are marked with thick boxes. Data of total score, social and nasal domain scores are depicted with mean ± standard deviation. Data of other scores are depicted with median values.

### Pituitary macroadenoma-related quality of life

There was no statistically significant difference in median age between patients with pituitary macroadenoma (48.6%, 58.8 years) and non-macroad-enoma (51.4%, 53.0 years) according to the Mann-Whitney U test (p > 0.05). Both groups did not statistically significantly differ in SBI-SLO total score and in all domains (p > 0.05), except in neurological domain, where there was a statistically significantly lower median score in non-macroadenoma group (63.7 points) than in macroadenoma group (84.3 points) according to Mann-Whitney U test (*U* = 239.000, *z* = 2.850, *p* = 0.004).

## Discussion

The management of diseases affecting the skull base, most notably tumours, necessitates a multidisciplinary approach that incorporates physicians from diverse specialties, along with members of the medical and non-medical professions. The patient’s subjective evaluation of their quality of life is also a pivotal factor in the treatment’s outcome.

The present study sets out the cross-cultural adaptation and validation of the Slovenian version of the SBI questionnaire. This instrument is intended for use in measuring the quality of life of Slovenian patients suffering from skull base disease. The results of the questionnaire revealed significant differences in quality of life between patients with malignant and benign skull base tumours. Furthermore, the study demonstrated that tumour type exerts a significant influence on the emotional and family aspects of quality of life.

It is evident from the extant research that the SBI is the most appropriate instrument for the assessment of quality of life in relation to disease in the anterior and central skull base, for both patients following open and endoscopic procedures. Consequently, it is a questionnaire that can be utilised in a broad spectrum of diagnoses and following a variety of procedures.^1^ Notwithstanding the aforementioned advantages of the SBI, to the best of our knowledge, the Slovenian cross-cultural validation and adaptation is the only non-English one that has been performed to date. This represents a significant milestone in the field of skull base pathology and patient-centered care. The SBI-SLO demonstrates excellent internal consistency (Cronbach’s alpha 0.92) and excellent test-retest reliability (ICC_A_ = 0.952), and permits adequate differentiation in quality of life between healthy volunteers and patients with skull base disease.^5,10^ Our analysis shows that SBI-SLO reached the ceiling in six domains, which means that domain scores of subjects are clustered at the highest extreme. This indicates that patients with maximum scores (i.e. 100 points) in these domains cannot be distinguished from each other. However, total SBI-SLO score did not reach the ceiling and none of the subjects achieved a maximum score (100). These results are consistend to the original SBI.^5^

Although concurrent validity analysis for every questionnaire translation is recommended^11^, it was not performed for SBI-SLO since there is no gold standard measurement instrument available for SBI^5^ and alternatives are not available in Slovene (e.g., Slovenian version of Anterior skull base questionnaire). However, concurrent validity has already been demonstrated in psychometric testing of an English SBI.^5^ Cross-cultural adaptation and validation of other questionaires and concurrent validity analysis presents a goal for future research.

In the present study, significant disparities in quality of life were identified between benign and malignant skull base tumour groups, with the latter exhibiting a comparatively diminished quality of life. Furthermore, the Cohen’s d value of 0.91 indicates a high effect size, signifying that the results possess significant practical relevance.^12^ The rule of thumb is that differences of 10% are clinically meaningful. Our study indicates possible clinically significant differences in quality of life between benign and malignant tumor groups, as the difference between them is 9.1 points on average, which represents 9.1%. To define the minimally important clinical difference, other quality of life indicators would also need to be taken into account, such as the global health-related quality of life change of “a little better” or “a little worse”.^5^ This represents a limitation of this study and, at the same time, an opportunity for further research.

The extant literature reports a significant reduction in quality of life in malignant tumours of the skull base^13^, but further research is required to determine the full extent of this phenomenon. The lower quality of life experienced by those afflicted with malignant tumours is also a consequence of the greater impact of the disease on both emotional state and family circumstances. The deleterious effect of malignant disease on emotional state has already been demonstrated^14^, thus corroborating the findings of the present study. Patients diagnosed with malignant tumours of the skull base could benefit from appropriate psychological or psychiatric treatment.^15^ The adverse impact of the tumour on family dynamics can manifest in various ways, including an increased reliance on relatives, a decline in the performance of family responsibilities, or a deterioration in interpersonal relationships among family members. The SBI and SBI-SLO are a measurement tools that can be utilised to assess these changes.^2^ The present study revealed a significant impairment in family relationships in the malignant tumour group. The impairment of family relationships has also been demonstrated in previous studies.^16,17^ Any cancer and, consequently, malignant diseases of the skull base can have far-reaching negative effects, as they also impair the quality of life of patients’ relatives, loved ones, and caregivers, and cause mental disorders such as depression.^17^

A notable distinction emerges between the age demographics of the patient cohort and the control group, which could potentially introduce a sampling bias, thereby challenging the ability to differentiate between the two groups. In order to eliminate this bias, it is imperative to have groups of the same age. Furthermore, the patient group encompasses a number of patients with metastases to the skull base, who may exhibit a generally poorer health status in comparison to other patient groups (e.g., pituitary macroadenomas). This phenomenon could be associated with a significant negative impact on specific domains of quality of life, including family life, emotional well-being, and other aspects. The study’s potential limitations stem from the relatively small number of patients, which resulted in a small sample size for specific types of benign and malignant skull base tumours. For instance, the quality of life in our group of benign tumours would be expected to be determined primarily by the quality of life of the subgroup of patients with pituitary macroadenomas, which predominate. However, the present study did not identify any significant disparities in quality of life between patients with pituitary macroadenomas and those with other benign tumours. The only discernible distinction pertains to the neurological domain, wherein pituitary macroadenomas generally do not manifest a clinical picture of neurological impairment (e.g., impairment of cranial nerves other than the optic nerve) in contrast to other benign tumours (e.g., petroclival meningiomas, craniopharyngiomas). The preponderance of pituitary tumours in our study is consistent with the findings of larger foreign studies.^18^ Moreover, the present study does not provide insight into the quality of life after treatment for skull base tumours, which is a goal for future research.

It should be noted that the present study does not include patients with lateral skull base tumours, which include vestibular schwannomas, as the SBI questionnaire is not an appropriate tool with which to assess quality of life in this disease. In order to surmount the limitations of the present study, it is imperative to adapt and cross-culturally validate an appropriate specific questionnaire for Slovenian.^19^

## Conclusion

A skull base tumour can exert a substantial detrimental effect on a patient’s quality of life, particularly in cases where it is malignant. The quality of life of the patient is an important indicator of the impact of the disease on patient’s experience of it. It is imperative to assess the quality of life in patients afflicted with such a debilitating disease, in order to deliver comprehensive care and achieve optimal treatment outcomes. The SBI is a key tool for assessing quality of life, and it is also available in Slovenian.

## Supplementary Material

Supplementary Material Details
