# Supplementary material for: Quality of life in Slovenian patients with skull base tumours: cross-cultural adaptation and validation of a Slovenian skull base inventory
Source: Radiol Oncol. 2026 Jan 21;60(1):106–13. doi: 10.2478/raon-2026-0007 (PMC13012383; doi:10.2478/raon-2026-0007)
Supplement: Supplementary file 1 — Supplementary Material Details [file raon-2026-0007_sm.pdf]

RADIOLOGY AND ONCOLOGY
Radiology and Oncology | Ljubljana | Slovenia | www.radioloncol.com

Quality of life in Slovenian patients with skull base tumours: cross-cultural adaptation and validation of a Slovenian skull base inventory

Domen Vozel, Jure Urbancic, Saba Battelino, Nina Bozanic Urbancic, Nejc Steiner, Tomislav Felbabic, Roman Bosnjak

doi: 10.2478/raon-2026-0007

Supplementary data 1

Quality of life questionnaire in cranial base disease in Slovenian (Slovenian skull base inventory, SBI-SLO)

VPRAŠALNIK O KAKOVOSTI ŽIVLJENJA PRI BOLEZNI LOBANJSKE BAZE (SBI-SLO)

Naslednjih 41 vprašanj nam pomaga razumeti, kako močno vas prizadene bolezen, ki se nahaja na lobanjski bazi. Svoje občutenje težav nam lahko poveste samo vi. Pričakujemo, da nam bodo rezultati vprašalnika pomagali bolje razumeti vašo bolezen. Prosimo, da natančno preberete in dobro razmislite na vsako zastavljeno vprašanje ter nanj odgovorite z prekrižanjem kvadratka poleg številke. Vsaka številka ustreza določeni stopnji (od 0 do 6). Napačnih ali nepravilnih odgovorov ni, a vseeno dobro premislite, preden izpolnite.

S podpisom izjavljam, da sem seznanjen/-a, da bo zdravstveno osebje v stiku s tem vprašalnikom upoštevalo novo evropsko zakonodajo o varstvu osebnih podatkov (Splošna uredba o varstvu podatkov-GDPR 2016/679 ter povezana Direktiva)

Če imate kakršnekoli težave z izpolnjevanjem vprašalnika, se obrnite na zdravstveno osebje za pomoč. Za odgovore se vam najlepše zahvaljujemo!

Začetnice imena in priimka:_________________ Datum rojstva:_______________

OBRNITE STRAN

Datum izpolnjevanja vprašalnika:_______________ **Koliko težav ste imeli v preteklih 2 tednih z... (vprašanja 1-13)**							
	Huda težava (0)	Večja težava (1)	Zmerna težava (2)	Občutna težava (3)	Minimalna težava (4)	Komajda težava (5)	Brez težav (6)
1. Glavobolom?	☐	☐	☐	☐	☐	☐	☐
2. Izcedkom iz nosu?	☐	☐	☐	☐	☐	☐	☐
3. Krastami v nosu?	☐	☐	☐	☐	☐	☐	☐
4. Dvojnim vidom?	☐	☐	☐	☐	☐	☐	☐
5. Vidnim poljem?	☐	☐	☐	☐	☐	☐	☐
6. Draženjem oči?	☐	☐	☐	☐	☐	☐	☐
7. Pogostim uriniranjem?	☐	☐	☐	☐	☐	☐	☐
8. Suhimi usti?	☐	☐	☐	☐	☐	☐	☐
9. Ohromelostjo in mravljinčenjem?	☐	☐	☐	☐	☐	☐	☐
10. Mišično oslabelostjo?	☐	☐	☐	☐	☐	☐	☐
11. Požiranjem hrane in pijače?	☐	☐	☐	☐	☐	☐	☐
12. Razdražljivostjo?	☐	☐	☐	☐	☐	☐	☐
13. Nezmožnostjo obvladovanja jeze?	☐	☐	☐	☐	☐	☐	☐

**Prosim ocenite naslednje zmožnosti in življenjske funkcije v zadnjih 2 tednih... (vprašanja 14-23)**							
	Brez (0)	Zelo slaba (1)	Slaba (2)	Ustrezna (3)	Dobra (4)	Zelo dobra (5)	Odlična (6)
14. Zaznava vonja	☐	☐	☐	☐	☐	☐	☐
15. Okus	☐	☐	☐	☐	☐	☐	☐
16. Dihanje skozi nos	☐	☐	☐	☐	☐	☐	☐
17. Jasen vid	☐	☐	☐	☐	☐	☐	☐
18. Raven energije	☐	☐	☐	☐	☐	☐	☐
19. Spanec	☐	☐	☐	☐	☐	☐	☐
20. Ravnotežje	☐	☐	☐	☐	☐	☐	☐
21. Spomin	☐	☐	☐	☐	☐	☐	☐
22. Zbranost	☐	☐	☐	☐	☐	☐	☐
23. Spolna funkcija	☐	☐	☐	☐	☐	☐	☐

**Kako močno zaskrbljeni ste bili v zadnjih 2 tednih zaradi... (vprašanja 24-27).**							
	Preveč (0)	Zelo močno (1)	Močno (2)	Zmerno (3)	Precej (4)	Malo (5)	Nič (6)
24. Videza telesa	☐	☐	☐	☐	☐	☐	☐
25. Telesne teže	☐	☐	☐	☐	☐	☐	☐
26. Sprememb v izgledu kože	☐	☐	☐	☐	☐	☐	☐
27. Hitrega nastanka modric	☐	☐	☐	☐	☐	☐	☐
28.							

Ali je v zadnjih 2 tednih vaše stanje ... (vprašanja 28—40)							
	Pre-<br>več (0)	Zelo<br>močno<br>(1)	Močn<br>o (2)	Zmer<br>no (3)	Prec<br>ej (4)	Malo<br>(5)	Nič (6)
29. Vplivalo na izvajanje aktivnosti	☐	☐	☐	☐	☐	☐	☐
30. Vplivalo na zmožnost druženja s prijatelji	☐	☐	☐	☐	☐	☐	☐
31. Vplivalo na delovne obveznosti	☐	☐	☐	☐	☐	☐	☐
32. Vplivalo na družinske obveznosti	☐	☐	☐	☐	☐	☐	☐
33. Postavilo odnose s prijatelji na preizkušnjo	☐	☐	☐	☐	☐	☐	☐
34. Vzbudilo dvom v veroizpoved	☐	☐	☐	☐	☐	☐	☐
35. Postavilo odnose v družini na preizkušnjo	☐	☐	☐	☐	☐	☐	☐
36. Povzročilo žalost ali depresijo	☐	☐	☐	☐	☐	☐	☐
37. Povzročilo zaskrbljenost za vaše zdravje	☐	☐	☐	☐	☐	☐	☐
38. Povzročilo frustracijo	☐	☐	☐	☐	☐	☐	☐
39. Povzročilo stres	☐	☐	☐	☐	☐	☐	☐
40. Povzročilo finančne izzive	☐	☐	☐	☐	☐	☐	☐
41. Povzročilo prekomerno odvisnost od drugih	☐	☐	☐	☐	☐	☐	☐

Kako bi ocenili ... (vprašanje 41)							
	Groz<br>no (0)	Zelo<br>slabo<br>(1)	Slabo<br>(2)	Pov-<br>prečno (3)	Do-<br>bro (4)	Zelo<br>dobro (5)	Odlič<br>no (6)
42. Vašo sposobnost ceniti majhne stvari v življenju	☐	☐	☐	☐	☐	☐	☐

Supplementary data 2

Scoring of Quality of life questionnaire in cranial base disease in Slovenian (Slovenian skull base inventory, SBI-SLO)

**Točkovanje vprašalnika SBI-SLO**				
Domena	Vprašanja	Največja možna vsota točk (A)	Vsota točk (B)	Preračunano število točk za domeno (= B/A x 100)
Kognitivna (K)	12, 13, 21, 22	24		
Čustvena (Č)	35, 36, 37, 38	24		
Družinska (D)	31, 34, 40	18		
Finančna (F)	30, 39	12		
Socialna (S)	23, 28, 29, 32	24		
Duhovna (Du)	33, 41	12		
Endokrina (En)	7, 25, 26, 27	24		
Nosna (N)	2, 3, 14, 16	24		
Nevrološka (Ne)	1, 9, 10, 20	24		
Vidna (V)	4, 5, 6, 17	24		
Ostalo (O)	8, 11, 15, 18, 19, 24	36		
Skupno število točk = seštevek preračunanih števil točk vseh domen				

Najnižje možno število točk je 0 in največje 100.

Supplementary data 3

Item-total statistics of Quality of life questionnaire in cranial base disease (Slovenian skull base inventory, SBI-SLO)

	Scale mean if item deleted	Scale variance if item deleted	Corrected item-total correlation	Squared multiple correlation	Cronbach's alpha if item deleted
Q1	170.58	1300.662	.463	.842	.922
Q2	170.49	1343.427	.215	.759	.925
Q3	169.46	1326.046	.382	.788	.923
Q4	169.29	1314.760	.360	.859	.923
Q5	169.81	1284.775	.491	.958	.922
Q6	169.75	1293.986	.541	.874	.921
Q7	169.68	1346.739	.228	.822	.924
Q8	170.56	1299.251	.500	.817	.922
Q9	169.05	1329.256	.375	.905	.923
Q10	169.53	1287.943	.656	.902	.920
Q11	169.27	1297.201	.548	.882	.921
Q12	169.27	1292.649	.635	.955	.920
Q13	168.69	1334.250	.429	.925	.922
Q14	170.58	1318.317	.346	.889	.924
Q15	170.22	1309.968	.431	.918	.922
Q16	170.31	1309.698	.497	.810	.922
Q17	170.61	1290.518	.588	.955	.921
Q18	170.66	1315.676	.500	.798	.922
Q19	170.61	1325.518	.431	.747	.922
Q20	170.10	1303.955	.655	.920	.920
Q21	169.71	1329.519	.418	.949	.923
Q22	169.83	1322.074	.524	.917	.922
Q23	171.34	1322.469	.380	.831	.923
Q24	169.29	1319.381	.449	.907	.922
Q25	169.08	1337.389	.330	.874	.923
Q26	168.85	1341.476	.349	.933	.923
Q27	168.49	1364.151	.205	.703	.924
Q28	170.41	1290.659	.568	.910	.921
Q29	169.88	1300.796	.511	.907	.922
Q30	170.51	1276.323	.615	.975	.920
Q31	170.10	1281.438	.578	.978	.921
Q32	169.00	1327.552	.388	.912	.923
Q33	168.49	1355.116	.205	.893	.924
Q34	168.81	1335.913	.403	.882	.923
Q35	169.73	1288.236	.699	.882	.920
Q36	170.97	1296.378	.609	.822	.921
Q37	169.58	1292.145	.567	.920	.921
Q38	170.14	1292.464	.533	.941	.921
